# Metabolic clock generates nutrient anticipation rhythms in mTOR signaling

**DOI:** 10.18632/aging.100686

**Published:** 2014-08-26

**Authors:** Rohini V. Khapre, Sonal A. Patel, Anna A. Kondratova, Amol Chaudhary, Nikkhil Velingkaar, Marina P. Antoch, Roman V. Kondratov

**Affiliations:** ^1^Center for Gene Regulation in Health and Diseases, BGES, Cleveland State University, Cleveland, OH 44115, USA; ^2^Department of Molecular Genetics, Cleveland Clinic, Cleveland, OH 44115, USA; ^3^Department of Molecular and Cellular Biology, Roswell Park Cancer Institute, Buffalo NY 14263, USA

**Keywords:** Food anticipation, metabolism, mTOR signaling pathway, biological clocks, circadian clocks

## Abstract

The mTOR signaling pathway modulates metabolic processes with respect to nutrient availability and other growth-related cues. According to the existing paradigm, mTOR complex 1 (mTORC1) activity *in vivo* is induced by food and gradually decreases during fasting. We found that mTORC1 activity is controlled by an internal clock mechanism different from the known light-entrainable circadian clock. We observed 24-hr rhythms in phosphorylation of mTORC1 downstream targets, which were entrained by food, persisted during fasting and could be uncoupled from oscillating expression of the canonical circadian clock genes. Furthermore, these rhythms were present in tissues of mice with disrupted light-entrainable circadian clock. We propose tissue-specific rhythms in the expression of *tor* and its negative regulator *deptor* as the molecular mechanism of the mTORC1 activity oscillation. Our data demonstrate the existence of at least two independent molecular circadian clocks: one providing metabolic adaptation to periodic light/darkness and the other - to feeding.

## INTRODUCTION

The mechanistic target of rapamycin (mTOR) signaling pathway senses and integrates information on availability of nutrients, growth factors and oxygen, evaluates the overall energy and stress status of the organism, and orchestrates anabolic and catabolic processes leading to cell growth, proliferation or death [1, 2]. Given the involvement of the mTOR pathway in pathophysiology of cancer, diabetes and aging [3], regulation of mTOR activity is extensively studied; however, up to date, the majority of work has been done in cell culture. Thus, physiological systems that control the mTOR pathway *in vivo* are still mostly unknown. Activity of the mTOR complex 1 (mTORC1) is associated with control of metabolism and is known to be stimulated by feeding. Indeed, food digestion results in secretion of insulin and temporal increase in concentration of amino acids - known positive regulators of mTORC1. According to the existing paradigm, under normal unstressed conditions mTORC1 activity mirrors the levels of its positive stimulators, and thus gradually decreases during fasting. Thus, activity of mTOR is currently thought to be regulated mostly by external stimuli rather than some internal system.

Many animals, and particularly mammals, demonstrate clear feeding rhythms in behavior: they eat during one part of the daily cycle and hide/rest/sleep during the other part of the day. Periodic availability of food and periodic feeding dictate adaptive behavioral and appropriate metabolic responses; one of the examples is the control of metabolic rhythms by the light-entrainable circadian clock. The light-entrainable circadian clock is, however, not the only internal system that regulates the feeding response. Indeed, food anticipatory (FA) rhythms in behavior are well documented [4, 5]. Although molecular mechanisms are still unknown, there is a significant body of evidence arguing that an internal oscillator, different from the known circadian clock, controls these FA rhythms. Periodic feeding suggests periodic supply of nutrients; therefore, existence of nutrient anticipation metabolic oscillators (NAMO) can be hypothesized: these oscillators would orchestrate metabolic processes in visceral organs in an analogous manner with the FA oscillator controlling the feeding behavior in the central nervous system. Studying anticipatory metabolic rhythms in visceral tissues is limited due to the lack of known molecular pathways involved. We hypothesized that because mTOR activity is regulated by nutrients, it may also be regulated by NAMO. In this case, nutrients would serve as external cues that allow entraining of the internal time-keeping metabolic system. We assayed activity of mTORC1 across the daily cycle and found that this activity is controlled by an internal oscillator different from the known light-entrainable circadian clock.

## RESULTS

**Figure 1:**
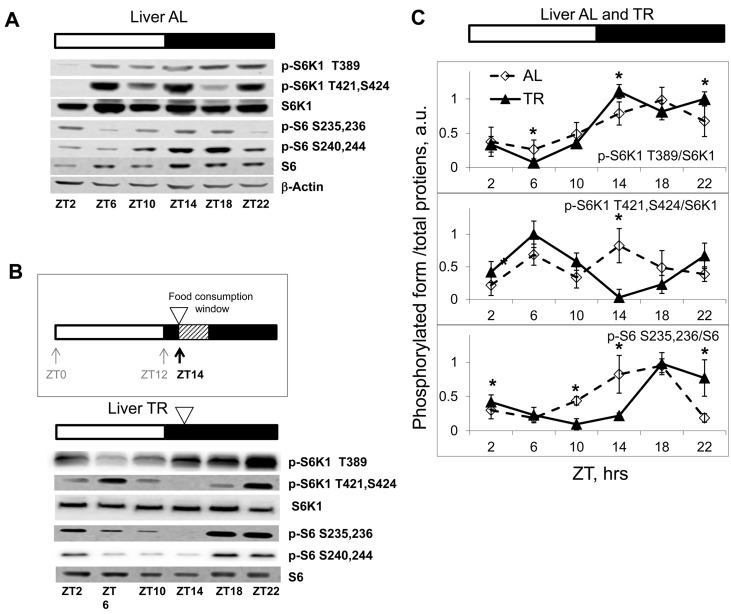
Daily rhythms in mTORC1in tissues of mice subjected to different feeding paradigms. Bars on the top of the figure represent the light (open bars) and the dark (black bars) parts of the day. ZT0 is the time when the light is turned on. (**A**, **B** and **C**) Representative Western blots (WB) of daily rhythms in phosphorylation of mTORC1 downstream targets: (**A**) in the liver of *ad libidum* (AL) fed mice; (**B**) in the liver of time restricted (TR) fed mice. The TR regimen is schematically depicted: 100% of the average daily ration was provided as a single meal at ZT14 (the white arrowhead); the food was consumed during three hours. (**C**) Quantification of phosphorylation of S6K1 and ribosomal S6 proteins on indicated sites normalized to total levels of the indicated proteins in the liver of AL (open diamonds, dashed black line) and TR (black triangles, solid black line) fed mice. 3 male mice per each time point and feeding regimen have been used. Data present Average +/− SEM; * p<0.05, statistically significant difference between different treatments.

We monitored mTORC1 activity by assaying phospho-rylation of its downstream targets: the ribosomal protein S6 kinase 1 (S6K1) and ribosomal protein S6 in the tissues of wild type mice. We found that the levels of phosphorylation of the mTORC1 downstream targets significantly changed during the day in the liver (Figure 1A). Levels of phosphorylation reached a peak during the dark phase of the daily cycle when mice were active (feeding), and dropped low during the middle of the light part of the daily cycle, when mice slept/rested (fasting), supporting the previously published observations on the regulation of mTORC1 by feeding. However, tissues collected at the same time of the day from different mice showed significant variability in the phosphorylation status of S6K1 and S6 (compare phosphorylation signals on Figure 1A (one set of male mice), Supplementary Figure 1 (two sets of male mice) and Supplementary Figure 2 (one set of female mice). Because tissues were collected from the *ad libitum* (AL) fed animals, one possible explanation of this inter-individual variability in phosphorylation was the difference in feeding habits of individual animals. To test this possibility and to reduce the potential influence of uncontrolled feeding time, we applied the time-restricted (TR) feeding schedule.

**Figure 2:**
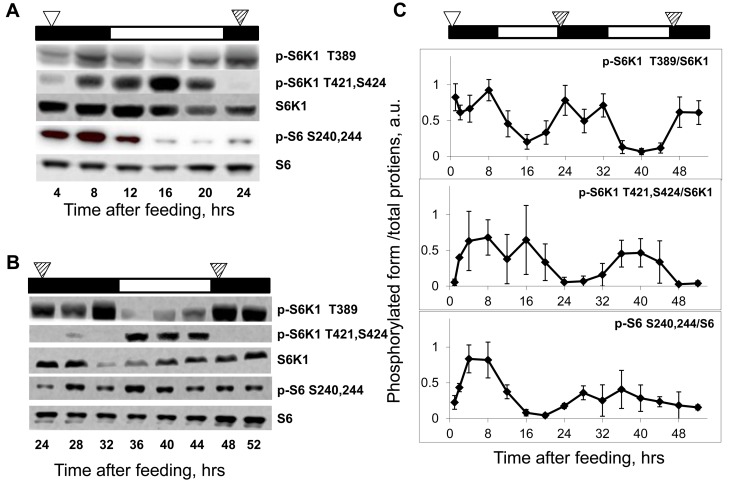
Daily rhythms in mTORC1 signaling are feeding-independent. Bars on the top of each figure represent the light (open bars) and the dark (black bars) parts of the day. (**A** and **B**) Representative western blots (WB) of daily rhythms in phosphorylation of mTORC1 downstream targets in the liver of TR fed mice. Food was provided at time point 0, and animals did not receive any more food throughout the experiment. (**A**) Animals have been sacrificed 4-24 hrs after the last feeding. (**B**) Animals have been sacrificed 24-52 hrs after the last feeding. The white arrowhead indicates the time of the last feeding; the striped arrowheads indicate the time of expected feeding (animals would expect the food, but the food was not provided). (**C**) Quantification of phosphorylation of S6K1 and ribosomal S6 proteins at indicated sites normalized to total levels of the indicated proteins in the liver of mice entrained by TR feeding. 3 male mice per each time point and feeding regimen have been used. Data present Average +/− SEM; * p<0.05, statistically significant difference between different treatments.

There are two main paradigms of time restricted feeding. In one approach, animals are placed in the cage containing food for a restricted period of time and then relocated to a food-free cage. In the other, the average daily food intake is determined, and 100% of the average daily ration is placed as a single meal at a particular time of the day (this approach is used for calorie restriction studies). To avoid additional stress associated with handling and re-location of the animals, we decided to use the second approach. Mice were caged individually and maintained on the 12 hr light/12 hr dark cycle; the food was offered at Zeitgeber Time (ZT) 14, when these nocturnal animals are normally active and eat (lights on at ZT0; lights off at ZT12). This TR regimen appeared to be physiologically relevant, since it did not cause detectable changes in locomotor activity of the animals (see later). No initial fasting and re-feeding was employed. After two weeks on this schedule, we observed that animals consumed the offered food during the first three hours; therefore, using this TR paradigm we were able to narrow the window of food consumption to 3 hrs and at the same time to avoid additional stress-inducing factors. As expected, TR feeding resulted in reduced inter-individual variability in phosphorylation of the mTORC1 downstream targets (Fig. 1B, see also Fig. 2A). In agreement with the known food-dependent induction, phosphorylation of the mTORC1 target S6K1 on T389 increased after feeding (maximum of phosphorylation was observed at ZT14-ZT22) and then gradually reduced to the minimum level (at ZT6) during the rest period. S6 is a downstream target of S6K1; in agreement with that, phosphorylation of S6 closely followed the phosphorylation pattern of S6K1 on the T389 site, with some delay. Interestingly, mTORC1-independent phosphorylation on Thr421,Ser424 sites under TR feeding showed two peaks during the daily cycle (an extra peak during the rest period). Taken together, these data demonstrate that there are daily rhythms in phosphorylation of the mTORC1 downstream targets (Figure 1C).

Importantly, we have noticed that after the level of phosphorylation of S6K1 at the T389 site reached its minimum at ZT6 (16 hrs after feeding), it then started to increase (Figure 1B and 1C). The signal at ZT10 (20 hours after the feeding) was higher than at ZT6 (16 hours after feeding) and was further increased at ZT14 (24 hrs after feeding, just before the next food administration), reaching a level comparable with the level at 4-8 hrs after feeding. Whereas the high level of phosphorylation at 4 and 8 hrs after feeding may present a direct response to feeding and increased level of nutrients and nutrient-induced insulin secretion, the increase in phosphorylation at 20 and 24 hrs after feeding (4 and 0 hrs before feeding) strongly suggests an internal regulation of these phosphorylation rhythms.

In an attempt to separate the effect of the external cue (food) from the potential internal mechanism, in the next set of experiments we assayed phosphorylation rhythms in animals that were maintained on the TR feeding regime for two weeks but did not receive food on the days of tissue collection; thus, mice were fasting for up to 52 hrs. As shown in Fig. 2A and 2B, phosphorylation levels of S6K1 T389 were the highest at 4-12 hrs, 24-32 hrs and 48-52 hrs, and the lowest at 16 hrs and 36-40 hrs after feeding. Thus, rhythms in phosphorylation of S6K1 in the liver of food-deprived animals were near 24 hrs in period and persisted for at least two cycles (three peaks/two troughs were observed for p-S6K1 T389) in the absence of the external cue.

Fig. 2C presents the quantitative comparison of phosphorylation rhythms in the liver of the TR feeding-entrained animals over the period of 52 hrs after the last feeding. Most likely, during the first cycle after feeding, mTORC1 activity (levels of phosphorylation) is affected by both external stimuli (food/nutrients) and internal mechanisms, while during the second and third cycles it is controlled only internally, which explains some differences observed between the first and the next cycles (see below). Taken together, these data strongly suggest that the rhythms in mTORC1 activity are synchronized to an external signal (feeding) and driven by an internal mechanism; we suggest to name this mechanism Nutrient Anticipation Metabolic Oscillator (NAMO). As mentioned above, the mTORC1-independent T421,S424 phosphorylation of S6K1 under TR conditions showed two peaks (Figure 2A): the first one at 4-8 hrs and the second one at 16 hrs after feeding. Under food-deprived conditions, only one peak at 36-40 hrs after feeding persisted (Figure 2B), but no elevation of phosphorylation was detected at 28-32 hrs after the last feeding, suggesting that the 4-8 hrs peak is driven by an external cue (food availability), while the second, 16 hrs peak, is internally driven (Fig. 2A and 2B). Of note, while phosphorylation of the mTORC1-indirect target S6 was rhythmic after feeding (Figure 2A), it did not demonstrate rhythms in the liver of food-deprived mice (Fig. 2B), indicating that regulation of phosphorylation of S6 *in vivo* may differ from the one *in vitro*, and should be used as an indicator of mTORC1 activity *in vivo* with caution.

**Figure 3:**
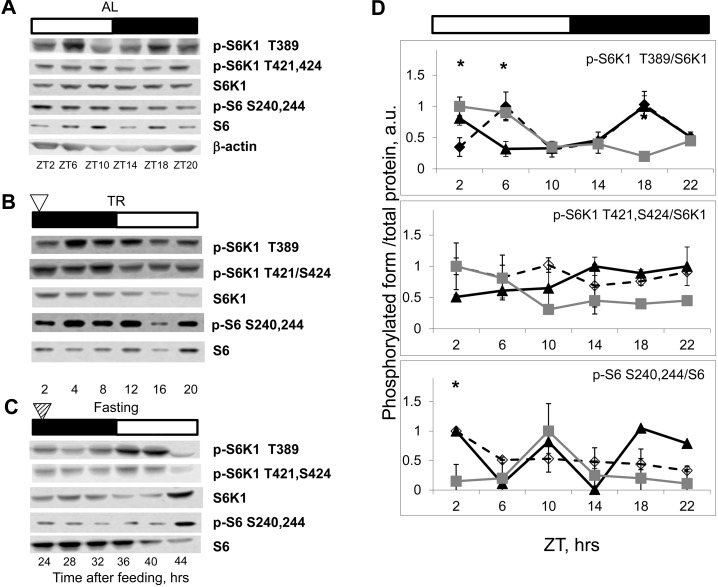
NAMO-controlled rhythms in mTORC1 activity are present in the spleen. Bars on the top of the figure represent the light (open bars) and the dark (black bars) parts of the day. ZT0 is the time when light is turned on. (**A**, **B** and **C**) Representative Western blots (WB) of daily rhythms in phosphorylation of mTORC1 downstream targets in the spleen: (**A**) of *ad libidum* (AL) fed mice; (**B**) of time restricted (TR) fed mice; (**C**) of fasted (F) mice (animals were entrained to TR feeding, 100% of daily food intake was provided as a single meal at the time point 0 (ZT14), but no food was provided for the last period (24-44h)). The white arrowhead indicates the time of feeding; the striped arrowhead indicates the time of expected feeding. (**D**) Quantification of phosphorylation of S6K1 and ribosomal S6 proteins on indicated sites normalized to total levels of the indicated proteins in the spleen of AL (open diamonds, dashed black line), TR (black triangles, solid black line) and F (gray squares, gray line) mice. 3 male mice per each time point and feeding regimen have been used. Data present Average +/− SEM; * p<0.05, statistically significant difference between different treatments.

The liver is an organ with high metabolic activity and is an important regulator of nutrient homeostasis. Due to its unique position in the digestive system, the liver is supplied with blood from the hepatic portal vein; therefore, the levels of nutrients and insulin during feeding and fasting can differ dramatically there and may have a direct strong effect on mTORC1 activity. Therefore, we decided to assay rhythms in mTORC1 activity in the spleen: the organ that is not a component of the digestive system and is exposed to the systemic circulation. As shown in Figure 3A (for males) and Supplementary Figure 3 (for females), phosphorylation of mTORC1 targets oscillated during the day in the spleen of AL fed mice. Similarly to the effect observed in the liver, TR feeding led to robust rhythms in phosphorylation in the spleen (Figure 3B), and these rhythms were preserved in the spleen of fasting animals (Figure 3C). Quantitative data for phosphorylation for the spleen are presented on Figure 3D. Maximum S6K1 T389 phosphorylation was 4-12 hrs and 36-40 hrs after feeding. Thus, similarly to the liver, we observed rhythms with near 24 hrs period in the spleen, and again during the first 24 hr cycle mTORC1 activity was controlled both by external stimuli and an internal oscillator, whereas in the following cycles it was controlled solely by internal mechanisms (i.e., the peak in S6K1 T389 phosphorylation observed at 4 hrs after feeding most likely resulted from the external stimulus, while peaks at 12 and 36 hrs after feeding were due to the internal regulation). Thus, we detected internally driven rhythms in activity of mTORC1 in organs from different systems, suggesting universality of the mechanism.

**Figure 4:**
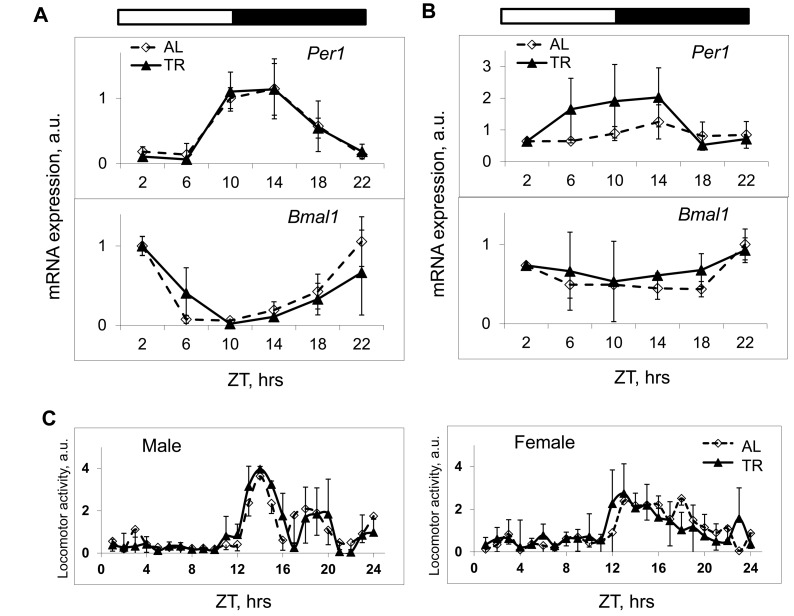
TR feeding does not affect daily rhythms in gene expression and behavior controlled by the circadian clock. (**A** and **B**) The expression of mRNAs of the circadian clock genes *Per1* and *Bmal1* in the liver (**A**) and in the spleen (**B**) of mice subjected to AL (black diamonds, solid black line) or TR (gray squares, gray line) feeding. (**C**) Daily in cage behavior of mice subjected to AL (black diamonds, solid black line) or TR (black triangles, dashed black line) feeding. 3 male mice per each time point and feeding regimen have been used. Data present Average +/− SEM; * − p<0.05, statistically significant difference between different treatments.

An interaction between the mTOR signaling pathway and the circadian clock has been recently hypothesized [5, 6]; in addition, an effect of feeding on circadian clock gene expression in the liver was demonstrated [7]. Because the NAMO-controlled rhythms in mTORC1 activity have the 24 hrs period and are synchronized by feeding, we decided to investigate if the circadian clock controls NAMO. We monitored the circadian rhythms in clock genes expression in the liver and spleen, and found that TR feeding did not affect amplitudes or phases of circadian rhythms in expression of *Bmal1*, *Per1* (Figure 4A) and several other circadian clock genes in the liver (Supplementary Figure 4) or circadian rhythms of *Bmal1* and *Per1* gene expression in the spleen (Figure 4B). We also monitored if TR feeding had any effect on daily locomotor activity. Because food was provided during the normal feeding time, as expected, we found no significant effect on daily behavior (Figure 4C and Supplementary Figure S1). Thus, the used TR feeding regimen did not disturb this major output of the circadian clock. At the same time, as shown above, we observed a significant effect of TR feeding on mTORC1activity in both liver and spleen (Figure 1, 2 and 3). These results suggest that the rhythms in mTORC1 signaling can be uncoupled from rhythms in circadian clock gene expression, and argue that NAMO and the light-entrainable circadian clock could be two independent internal rhythm-generating systems.

**Figure 5:**
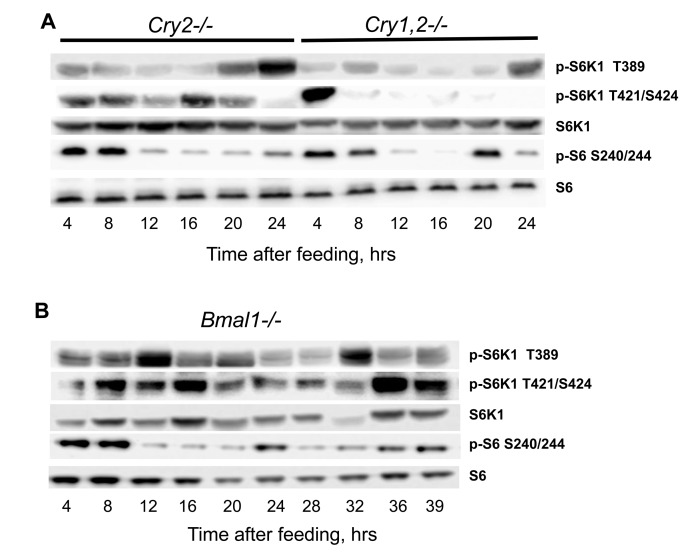
NAMO controlled rhythms in TORC1 activity are preserved in the tissues of circadian clock deficient mice. (**A** and **B**) Representative western blot (WB) of daily rhythms in phosphorylation of mTORC1 downstream targets in the liver of TR feeding entrained mice with circadian clock disruption. 100% of daily food intake was provided as a single meal at time point 0 (ZT14), tissues were collected at indicated time after feeding (**A**) *Cry2^−/−^* and *Cry1,2^−/−^* mice, as indicated; (**B**) *Bmal1^−/−^* mice.

In order to directly test if NAMO is different from the known circadian clock, we assayed rhythms in mTORC1 activity in the tissues of mice deficient for the core components of the circadian clock: *Cry1*, *Cry2* and *Bmal1*. Circadian clock deficient mice have been entrained by TR feeding, and tissues were collected around the 24-hrs cycle. *Cry1* deficiency shortens the period of rhythms, *Cry2* deficiency leads to period elongation, and double deficiency of these genes results in complete disruption of circadian rhythms in gene expression and behavior [8]. Neither deficiency for *Cry2* nor double deficiency for both *Cry1* and *Cry2* affected rhythms in phosphorylation of S6K1 on the mTORC1-dependent T389 site: the minimum was observed around 12-16 hrs after feeding and the maximum - at 24 hrs or just before the feeding similarly to wild type (Figure 5A). Interestingly, the rhythms in phosphorylation on the mTORC1-independent sites 421,424 were not affected by deficiency of *Cry2*: similarly to wild type there were two peaks during the 24h period, the first peak at 4 hrs after feeding and the second at the middle of the light phase (16 hrs after feeding); however, although the first, food-dependent peak was preserved in the liver of *Cry1,2^−/−^* mice, the second peak was absent (Figure 5A), suggesting that this peak may be controlled by the light-entrainable circadian clock. We also assayed rhythms in the liver of *Bmal1^−/−^* deficient mice (Figure 5B). Importantly, we did not observe the peak in T389 phosphorylation at 24-28 hrs, but we detected a peak at 32-hrs after feeding.

Thus, rhythms in T389 phosphorylation were still present in *Bmal1^−/−^* mice, but these rhythms were different from the rhythms in wild type or other circadian mutants. Previously we reported that mTORC1 signaling is disrupted in *Bmal1−/−* mice. [9]. Thus, BMAL1 might be involved in both light-entrainable circadian clock and NAMO; interestingly, premature aging phenotype is characteristic to *Bmal1* deficiency only and not associated with deficiency of other circadian clock genes studied so far, which suggest that BMAL1 function in control of metabolism and aging cannot be explained through its light-entrainable clock functions. Thus, data presented on Figures 4 and 5 argue that rhythms in mTORC1 signaling are controlled by NAMO and are independent from the canonical light-entrainable circadian clock.

**Figure 6:**
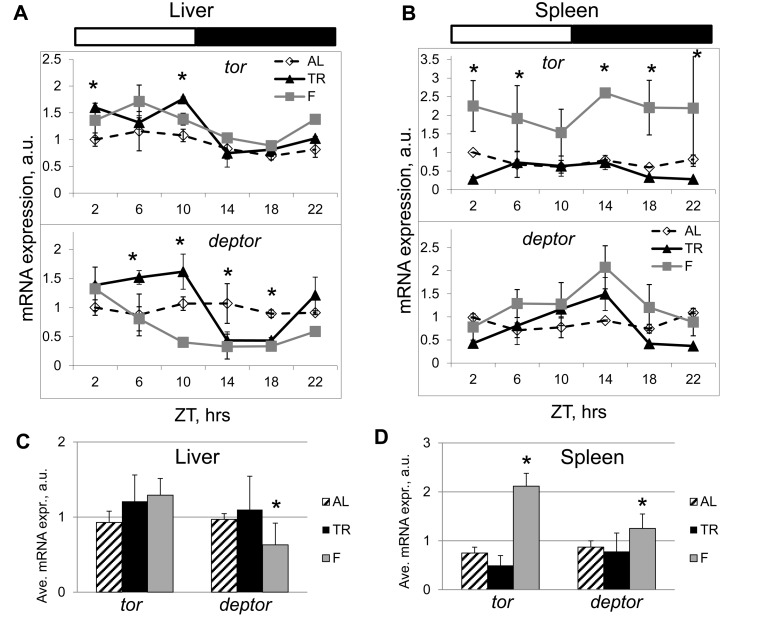
TR feeding results in rhythmic expression of mTORC1 components. Daily profiles of mRNA expression of *tor* and *deptor* in (**A**) the liver and (**B**) the spleen of mice subjected to AL (open diamonds, dashed black line), TR (black triangles, solid black line) and F (gray squares, gray line). The same animals have been analyzed on Figure 1, 2 and 3 for mTORC1 signaling. ZT0 is time when light turn on. (**C** and **D**) Average daily expression of *tor* and *deptor* in the liver (**C**) and in the spleen (**D**): AL (striped bars), TR (black bars), F (gray bars). 3 male mice per each time point and feeding regimen have been used. Data present Average +/− SEM; * − p<0.05, statistically significant difference between different treatments.

In order to get an insight on possible mechanisms of mTOR pathway regulation by NAMO, we assayed expression of the components of mTORC1 at the mRNA level. TR feeding significantly affected expression of the *tor* kinase and of the mTORC1component *deptor* (which is a negative regulator of mTORC1) in the liver (Figure 6A) and spleen (Figure 6B) of wild type mice. While the expression of *tor* in AL fed mice was rhythmic with low amplitude in the liver (Figure 6A, upper panel) and arrhythmic in the spleen (Figure 6B, upper panel), the expression was rhythmic in both tissues in TR animals (Figures 6A and B, upper panels). Similar to that, expression of *deptor* was rhythmic in the liver of AL fed animals with a relatively small amplitude and arrhythmic in the spleen, while TR feeding resulted in rhythmic daily expressions of *deptor* both in the liver and spleen with a significant amplitude, and in a shift of the phase of *deptor* expression in the liver (Figures 6A and 6B, lower panels). Importantly, the positive (*tor*) and negative (*deptor*) regulators of mTORC1 had different phases of expression, which can provide a mechanistic base for the observed rhythms in mTORC1 activity.

To dissect whether the observed rhythms in expression of mTORC1 components resulted from the rhythmic supply of nutrients or from an internal regulation, we assayed their expression in the liver and spleen of mice that were subjected to fasting. Rhythms in expression induced by TR feeding persisted under these constant (fasting) conditions (Figure 6A and B); however, similarly to the observed difference between the first and next cycles in mTORC1 target phosphorylation (Figure 2), we observed a specific difference between TR and fasting rhythms. Maximums of expression of *tor* and *deptor* in TR samples were more prolonged than in fasting samples, suggesting that rhythms in TR arise from a combination of feeding/nutrient-dependent and internally regulated mechanisms. These results are in striking contrast to the effects of TR feeding on the expression rhythms of clock genes (Figure 4). The expression rhythms of the circadian clock genes had small amplitudes in the spleen and high amplitudes in the liver; in both cases the rhythms were not affected by TR feeding (Figure 4). On the contrary, TR feeding resulted in a great increase in the amplitude of rhythms in the expression of *deptor*: the amplitudes were equally high in both tissues (Figures 6A and 6B). Interestingly, in fasting animals, phases of the expression rhythms for *tor* were different between the liver (the maximum was at ZT6) and the spleen (the maximum was at ZT14), the same was true for *deptor*: expression in the liver had the maximum at ZT2 and in the spleen at ZT14. The phase shift in the expression between the liver and spleen is in an agreement with different phases of mTORC1 activity in these tissues (again in a contrast to the same phases of circadian clock gene expressions in the liver and spleen). Thus, rhythms in *deptor* and *tor* expression induced by TR feeding are uncoupled from rhythms in the circadian clock gene expression, which further supports the difference between the light-entrainable circadian clock and NAMO. We also calculated the average daily expression of *tor* and *deptor* under different conditions. In the liver (Figure 6C) and spleen (Figure 6D), TR did not affect the daily average for *tor* and *deptor*, thus, the effect of periodic feeding/nutrient supply resulted mostly in a redistribution of expression across the day. Fasting significantly affected the daily average and distribution of expression for *deptor* in the liver and expression of *tor* in the spleen, again suggesting that expression of these genes is under control of internal and external regulators.

## DISCUSSION

**Figure 7:**
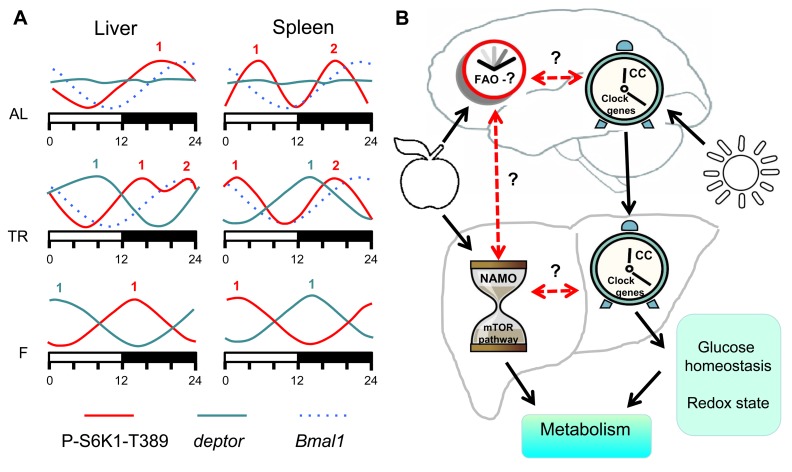
Model of metabolism control by NAMO and the circadian clock. (**A**) Feeding regimens affects NAMO but not the light-entrainable circadian clock. Schematic representation of daily changes in the expression of circadian clock controlled gene *Bmal1* (dashed blue line), *deptor* (solid blue line) and phosphorylation of S6K1 on mTORC1-dependent T389 site (solid red line). AL (*ad libidum*), TR (time restricted) fed mice and F (fasting) mice. Numbers indicate peaks in the expression activity. Bmal1 expression has the same phase in both tissues under all three feeding regimens, while phases in the expression of *deptor* and mTORC1 activity are different in different tissues and for different feeding regimens. (**B**) The light-entrainable circadian clock and NAMO regulate metabolism through complementary pathways. In the brain there are two clock mechanisms: food anticipatory (FA) clock and light- circadian clock (CC). In the body there are also two clocks: NAMO and CC. Daily rhythms in NAMO dependent regulation of TORC1 and CC dependent regulation of glucose homeostasis and cell redox state will contribute to daily control of metabolism. Brain CC coordinates activity of CCs in the body. NAMO and FAO are entrained by feeding. If these three clock mechanisms (CC, NAMO and FAO) interact and coordinate their activities or not is unknown, but some experimental evidence exists that such interaction is possible. Solid black lines indicate established interaction and dashed red lines indicate potential interaction.

There are many oscillating processes in biology such as the cell cycle, daily cycle, reproduction cycle and developmental cycle. Daily rhythms in behavior and physiology of living organisms are well known; some of these rhythms are driven by external cues and others are controlled by internal mechanisms. One of the systems regulating daily activity is the circadian clock. The circadian clock is the light-entrainable genetically determined system composed of several interdependent transcription-translation feedback loops, which generates periodicities in sleep, locomotor activity and metabolism known as the circadian rhythms. The circadian clock is not the only internal clock that generates rhythms with a near 24-hr periodicity: rhythms in food anticipatory (FA) behavior is one of the best known examples of 24-hr rhythms generated independently from the light-entrainable circadian clock; moreover, a transcription-independent circadian (24h-periodical) clock was recently reported to control the cellular redox state [10]. Transcription-translation feedback loops comprising the known circadian clock in animals and other organisms have been discovered about two decades ago; however, molecular components of food-entrainable oscillators are still unknown, which imposes significant limitations on the research of these oscillators. Recently, it was proposed that FA clocks in peripheral organs and in the CNS interact in order to generate the net FA activity [4]. Using the time restricted feeding paradigm, we found that daily rhythms in mTORC1 activity are under control of an internal food-entrainable oscillator, which we named NAMO. We found that NAMO is not identical to the known light-entrainable circadian clock. Indeed, the experimental approach used in our study let us to uncouple the rhythms in mTORC1 activity from the circadian rhythms in expression of the canonical clock genes. Figure 7A summarizes the effects of different feeding regimens on NAMO- and the circadian clock-controlled rhythms in different tissues. Rhythms in circadian clock gene expression in the liver and spleen had the same phase and were not influenced by different feeding regimens (i.e., maximum of *Bmal1* expression was reached at the same time), on the other hand, rhythms in mTORC1 activity were significantly shifted by the time-restricted feeding, and phases of these rhythms were different in different tissues. Under conditions of TR feeding, we observed two maximums in the liver (ZT14 and ZT22), while under fasting conditions - only one maximum at ZT14, which was different from the AL maximum at ZT18. We propose that the internal oscillator produces the ZT14 maximum, which is preserved upon fasting, while the ZT22 maximum is associated with the external feeding cue. The less pronounced ZT18 maximum detected under the AD conditions could be thus a combination of the internal maximum and multiple local peaks in mTOR activity resulting from AD food consumption. Recently, an interaction between the circadian clock and the mTOR signaling pathway has been proposed [5, 6]; however, our findings that the rhythms in mTORC1 activity are preserved in the tissues of circadian clock deficient mice clearly demonstrate that these rhythms are under control of a different, independent internal oscillator - NAMO. Importantly, in a strong contrast to expression of clock genes, expression of *tor* and *deptor* was dramatically affected by rhythmic feeding; moreover, in agreement with the shift in mTORC1 activity, expression of *tor* and *deptor* was phase-shifted between the liver and the spleen, while circadian genes expressed with the same phases in both organs.

These rhythms in transcriptional control of the expression of the mTORC1 components might provide a molecular mechanism of NAMO. Indeed, in addition to regulation by external stimuli, activity of the mTOR complex1 (mTORC1) is controlled by multiple positive and negative feedback loops. Examples of such regulation are the mTORC1-S6K1-IRS1 negative feedback loop or the mTORC1-Deptor loop [3]. Thus, similarly to other oscillatory mechanisms known to be formed by inter-acting feedback loops, such as the light-entrainable circadian clock, feedback loops in the mTORC1 pathway and mTORC1-dependent regulation of transcription and translation may constitute the basis for the observed food-independent internal rhythms in mTORC1 activity *in vivo*.

What is the biological significance of the feeding-independent component of the rhythms in the control of mTOR signaling? One potential explanation is that the organism activates and synchronizes all anabolic processes in the expectation of food in order to digest and stored all received nutrients in the most efficient way. It was proposed that the circadian clock-dependent control of glucose or fat metabolism serves for the same propose. Whether the light-entrainable (the circadian clock) and the food-entrainable (NAMO) clocks have overlapping (redundant) or complimentary functions, has to be found in future studies. Another potential role of NAMO is the equally important control of catabolism. According to the existent paradigm, mTORC1 activity increases upon feeding in response to the raise of the levels of amino acids and release of some growth factors in the bloodstream, while during the period of starvation mTORC1 activity gradually decreases as nutrients are being consumed and growth factors drop in concentration [3]. However, it is well-known that multicellular organisms, especially mammals, support a nearly constant environment for their cells in tissues, therefore regulation of mTORC1 activity only by circulating amino acids and growth factors can be insufficient. Taking into account that daily changes in amino acids and growth factor concentrations *in vivo* are modest (about 20% in amplitude) [11] [12, 13], compared with the complete withdrawal in cell culture (where the majority of observations about regulation of mTORC1 were made), the significant and sharp reduction of mTOR activity that we observed *in vivo* would seems paradoxical if this activity was regulated by nutrients/growth factors concentrations in a passive way. Our data strongly suggest the existence of an internal mechanism for the active suppression of mTORC1.

Importantly, when we compared rhythms in mTORC1 activity in the liver and spleen, we observed a phase shift between these two tissues; the shift was especially pronounced under fasting conditions. Indeed, as it was discussed above, while after feeding the activity of mTORC1 is regulated by both external stimuli and internal mechanisms, under the fasting conditions only internal mechanisms work. This shift has a clear physiological significance: indeed, even under conditions of fasting the organism cannot avoid anabolic processes including cell growth and proliferation in the tissues with high levels of cell turnover. If all tissues initiate anabolic processes at the same time, they would compete for the same resources (nutrients) released by the liver and adipose tissue, which can result in energetic collapse and have a deleterious effect on physiological activity. Shifting phases of anabolic activities allows redistributing resources in a more efficient way. The prediction from this hypothesis is that if the circadian clock plays an important role in nutrient accumulation, NAMO activity is critically important to prevent overuse of resources at the time of starvation.

It is also important to assay whether FA rhythms in behavior, which are independent from the light-entrainable circadian clock [14], are mediated or modulated by NAMO, or whether another oscillator responsible for generation of FA rhythms does exist. This question is also linked with the question about anatomical location of NAMO (note that anatomical location of the FA center is still unknown [14]: is it in the central nervous system, in visceral organs or in both? Systemic mechanisms suggest periodic changes in concentration of some factor(s). One of the most obvious candidates is insulin: it is known that insulin can be released in the absence of food in the expectation of feeding (Pavlovian conditioning) and can be provoked by stimuli associated with food expectation (noise from locomotor activity produced by neighbor mice expecting the food). In other words, food anticipatory activity (clock-independent) may stimulate locomotor activity, which would lead to increased noise in the facility, in turn activating conditioning memory known to result in the secretion of insulin. While this scenario still needs to be tested in direct experiments, there are several facts observed in our study that argue that some additional mechanisms must exist. First, the used feeding regimen did not affect the pattern of daily behavior, therefore, no extra noise providing a conditioning cue was generated; second, mTORC1 activity started to increase about 4 hrs before the feeding, which is too early to be a response to conditioning; third, mTORC1 activity remained high for several hours, while Pavlovian conditioning results only in a short spike of insulin release. Finally, mTORC1 activity in different tissues had different phases; to achieve this difference, response to external stimuli must be differentially regulated. Therefore, if NAMO activity in the liver and other visceral tissues is regulated by insulin or other factors secreted upon a command from an oscillator in the central nervous system (an FA center in the brain), this memory is not identical to the conditioning memory.

We propose the following model (Figure 7B): a food-entrainable NAMO regulates the mTOR signaling pathway and biosynthesis; the brain-located light-entrainable circadian clock coordinates metabolic activities such as glucose or redox homeostasis of peripheral circadian clocks. Joint activities of these two oscillators generate 24 hr rhythms in metabolism. In many cases periodic feeding is connected to periodic daily activity, which in turn is connected to the day/light cycle. Thus, activities of these two oscillators are probably synchronized and complement each other. For example, the light-entrainable system serves as a referent (“absolute”) clock, aligned with nearly constant astronomical conditions, while the food-entrainable system can mark a “relative” time when food is available in the current circumstances. Important questions that remain unanswered are whether and how these two oscillators interact. The following data argues in favor of such interaction: shifting the feeding time shifts the circadian clock in the liver and some other visceral organs without shifting the clock in the brain [7]. It was shown that mTOR signaling regulates the circadian clock [6]; thus, NAMO may signal to the circadian clock through mTORC1. Therefore, NAMO and peripheral circadian clocks can be synchronized to each other. A potential candidate enabling this connection is BMAL1, a component of the circadian clock [15] and a potential component of NAMO. Indeed, in contrast to other circadian mutants, the mTORC1 activity rhythms were significantly changed in tissues of *Bmal1*-deficient mice (Figure 5B). Importantly, although disruption of the circadian clock may affect the rate of aging, only deficiency of BMAL1 results in severe premature aging [16], while deficiencies of other circadian clock genes result in very mild aging phenotypes. One possible explanation is that activities of both food- and light-entrainable oscillators are important for the control of aging. Deficiency of BMAL1 affects both oscillators, while deficiency of other circadian clock proteins affects only the circadian clock but not NAMO.

In conclusion, in this study we show that a specialized food-entrainable internal oscillator (NAMO) that is different, but potentially interconnected, with the light-entrainable circadian clock, regulates activity of the mTOR pathway. We propose BMAL1 as a mediator of the two clocks, which allows explaining the unique accelerating aging phenotype associated with BMAL1 deficiency.

## MATERIALS AND METHODS

### Animals and tissue collection

*Bmal1^−/−^* mice were obtained from Dr. C. Bradfield (University of Wisconsin). *Cry1,2^−/−^* were obtained from Dr. A. Sancar (University of North Carolina at Chapel Hills). Animals were maintained on a 12:12 light:dark cycle with lights on at 7:00 am, and fed on an 18% protein rodent diet (Harlan). Ad libitum (AL) group had unrestricted access to food. Time restricted feeding (TR) group received about 100% of their daily intake at ZT14, mice have been entrained for TR for 2 weeks. Fasting group has been entrained to TR and did not receive the food during the experiment. All groups have unrestricted access to water. All tissue collection experiments have been performed for 3-4 months old wild type and circadian mutant mice. Daily in cage behavior was monitored using infrared photobeam in cage activity system (San Diego Instruments). Animal activity was recorded every 60 minutes during 4 days, and analyzed using Cage rack Software. Activity of the same mouse was recorded for AL feeding and after 14 days of TR. All animal studies were conducted in accordance with the regulations of the Committee on Animal Care and Use at Cleveland State University.

### Statistical analysis

At least 3 animals for every time point, for all feeding types and for each genotype (only male mice were used in all experiments, female mice were used only for one experiment shown on Supplementary Figure 2) were used for all experiments. Data are shown as mean +/− standard deviation. SigmaStat software package was used for analysis. P<0.05 was considered as statistically significant.

### Analysis of protein phosphorylation and expression

Tissue and cell extracts were prepared in RIPA buffer with Protease/Phosphotase Inhibitor Cocktail, Cell signaling Technology (#5872) according to the manufacturer's protocol. Tissue and cell extracts were normalized by measuring total protein concentration using Bio-Rad Dc protein Assay kit according manufacturer's protocol. Extracts were separated by SDS-PAGE, transferred on Immobilon-P membrane (Millipore) and incubated with phosphospecific and protein specific primary and secondary antibodies. Following antibodies were used: Cell Signaling Technology: Phospho-p70 S6K (Thr389) #9205, Phospho-p70 S6K (T421/S424) #9204, p70 S6K #9202, Phospho-S6 (Ser235/236) 32211, Phospho-S6 (Ser240/244) #2215, Anti-Mouse IgG-HRP #7076, aAnti-rabbit IgG-HRP #7074. Santa Cruz Biotechnology: Robosomal Protein S6 sc-74459, Donkey anti-rabbit IgG-HRP sc-2313. Abcam: Anti-S6K1 (phosphor T389) ab2571.

### Analysis of mRNA expression

Use the “Insert Citation” button to add citations to this document. Total RNA was isolated from with TriZol reagent (Invitrogen) according to the manufacturer's protocol. RNA quantization was performed using TaqMan real-time RT-PCR with CyberGreen mix (BioRad), relative mRNA abundance was calculated using the comparative delta-Ct method with 18S RNA. Sequence of primers can be found in Supplementary Information.

## SUPPLEMENTARY INFORMATION AND FIGURES


