# Supplementary material for: Metabolic clock generates nutrient anticipation rhythms in mTOR signaling
Source: Aging (Albany NY). 2014 Aug 26;6(8):675–89. doi: 10.18632/aging.100686 (PMC4169861; doi:10.18632/aging.100686)
Supplement: Supplementary file 1 [file aging-06-675-s001.pdf]

SUPPLEMENTARY INFORMATION

Experimental Procedures

Animals: Wild type and knock out mice were generated by breeding of heterozygotic parents. Genotypes were determined using a PCR-based method as previously described (Bunger et al., 2000; Vitaterna et al., 1999). Animals were maintained on a 12:12 light:dark cycle with lights on at 7:00 am, and fed on an 18% protein rodent diet (Harlan). Animals were maintained on a 12:12 light:dark cycle with lights on at 7:00 am, and fed on an 18% protein rodent diet (Harlan). Ad libitum (AL) group had unrestricted access to food. Time restricted feeding (TR) group received about 100% of their daily intake at zt14, mice have been entrained for TR for 2 weeks. Fasting (F) group were entrained to TR for two weeks but did not receive the food during the experiments for up to 52 hrs. All groups have unrestricted access to water. All tissue collection experiments have been performed for 3-4 months old wild type and mutant mice. Daily in cage behavior was monitored using infrared photobeam in cage activity system (San Diego Instruments). Animal activity was recorded every 60 minutes during 4 days, and analyzed using Cage rack Software. Activity of the same mouse was recorded for AL feeding and after 14 days of TR. All animal studies were conducted in accordance with the regulations of the Committee on Animal Care and Use at Cleveland State University.

Statistical analysis: At least 3 animals for every time point, for both feeding types and for each genotype were used for all experiments. Data are shown as mean + standard deviation. SigmaStat software package was used for analysis. Effects of genotype (circadian mutants versus wild type), feeding type (AL versus TR) (AL versus F) and time of the day were tested for significance. Unpaired Student's t-test was used for comparison. $P<0.05$ was considered as statistically significant difference.

Analysis of protein phosphorylation and expression: Tissue and cell extracts were prepared in RIPA buffer with Protease/Phosphotase Inhibitor Cocktail, Cell signaling Technology (#5872) according to the protocol described in (Kondratov et al., 2003). Tissue and cell extracts were normalized by measuring total protein concentration using Bio-Rad Dc protein Assay kit according manufacturer's protocol. Extracts were separated by SDS-PAGE, transferred on Immobilon-P membrane (Millipore) and incubated with phospho-specific and protein specific primary and secondary antibodies. Following antibodies were used: Cell Signaling Technology: Phospho-p70 S6K (Thr389) #9205, Phospho-p70 S6K (T421/S424) #9204, p70 S6K #9202, Phospho-4E-BP1 (T37/46) #2855, 4E-BP1 #9452, Phospho-S6 (Ser235/236) 32211, Phospho-S6 (Ser240/244) #2215, Anti-Mouse IgG-HRP #7076, aAnti-rabbit IgG-HRP #7074. Santa Cruz Biotechnology: Robosomal Protein S6 sc-74459, Donkey anti-rabbit IgG-HRP sc-2313. Abcam: Anti-S6K1 (phosphor T389) ab2571.

Analysis of mRNA expression: Total RNA was isolated from with TriZol reagent (Invitrogen) according to the manufacturer's protocol. RNA quantization was performed using TaqMan real-time RT-PCR with CyberGreen mix (BioRad), relative mRNA abundance was calculated using the comparative delta-Ct method with 18S RNA as standard as described in (Kondratov et al., 2006). Following primers have been used for the analysis of expression:

18s rRNA F 5' GCT TAA TTT GAC TCA ACA CGG GA 3'
18s rRNA R 5' AGC TAT CAA TCT GTC AAT CCT GTC 3'
PER1 F 5' AGG TGG CTT TCG TGT TGG 3'
PER1 R 5' CAA TCG ATG GAT CTG CTC TGA 3'
BMAL1 F 5' CCA AGA AAG TAT GGA CAC AGA CAA A 3'
BMAL1 R 5' GCA TTC TTG ATC CTT CCT TGG T 3'
MTOR F 5' ATT CAA TCC ATA GCC CCG TC 3'
MTOR R 5' TGC ATC ACT CGT TCA TCC TG 3'
DEPTOR F 5' GTG GTT CTC AGG CAT TCT ATC TC 3'
DEPTOR R 5' TGG GTA GGT TTT GAG ATG GTG 3'
RHEB F 5' AAG ATG CCT CAG TCC AAG TC 3'
RHEB R 5' CGT GTT CTC TAT GGT TGG ATC G 3'

References

1. Bunger MK, Wilsbacher LD, Moran SM, Clendenin C, Radcliffe LA, Hogenesch JB, Simon MC, Takahashi JS, and Bradfield CA. Mop3 is an essential component of the master circadian pacemaker in mammals. Cell. 2000; 103:1009-1017.

2. Kondratov RV, Chernov MV, Kondratova AA, Gorbacheva VY, Gudkov AV, and Antoch MP. BMAL1-dependent circadian oscillation of nuclear CLOCK: posttranslational events induced by dimerization of transcriptional activators of the mammalian clock system. Genes Dev. 2003; 17:1921-1932.

3. Kondratov RV, Shamanna RK, Kondratova AA, Gorbacheva VY, and Antoch MP. Dual role of the CLOCK/BMAL1 circadian complex in transcriptional regulation. FASEB J. 2006; 20:530-532.

4. Vitaterna MH, Selby CP, Todo T, Niwa H, Thompson C, Fruechte EM, Hitomi K, Thresher RJ, Ishikawa T, Miyazaki J, Takahashi JS, and Sancar A. Differential regulation of mammalian period genes and circadian rhythmicity by cryptochromes 1 and 2. Proc Natl Acad Sci U S A. 1999; 96: 12114-12119.

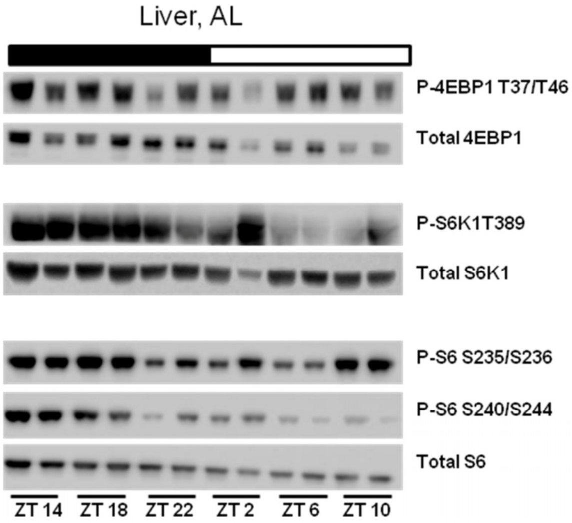

Figure S1. Phosphorylation and total levels of mTORC1 downstream targets S6K1, 4EBP1 and ribosomal S6 proteins in the liver of AL fed male mice through daily cycle. Immunoblotting on tissue collected at the indicated time of the day and probed with antibodies specific for indicated sites. For every time point tissue extracts from two different animals are shown. Bars on the top of the figure represent light (open bars) and dark (black bars) parts of the day.

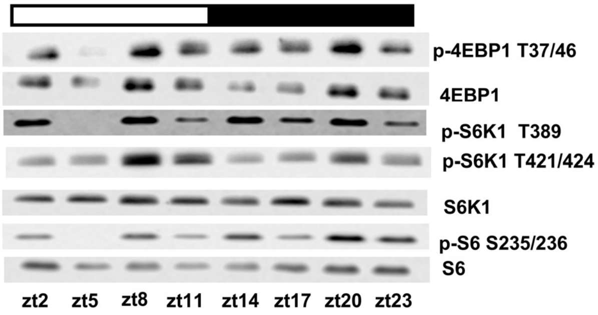

Figure S2. Phosphorylation and total levels of mTORC1 downstream targets S6K1, 4EBP1 and ribosomal S6 protein in the liver of AL fed female mice throughout the daily cycle. Immunoblotting on tissues collected at the indicated time of the day and probed with antibodies specific for indicated sites. Bars on the top of the figure represent light (open bars) and dark (black bars) parts of the day.

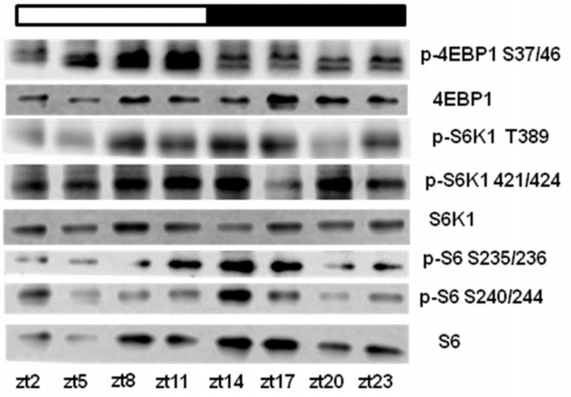

Figure S3. Phosphorylation and total levels of mTORC1 downstream targets S6K1, 4EBP1 and ribosomal S6 protein in the spleen of AL fed female mice throughout the daily cycle. Immunoblotting on tissue collected at the indicated time of the day and probed with antibodies specific for indicated sites. Bars on the top of the figure represent light (open bars) and dark (black bars) parts of the day.

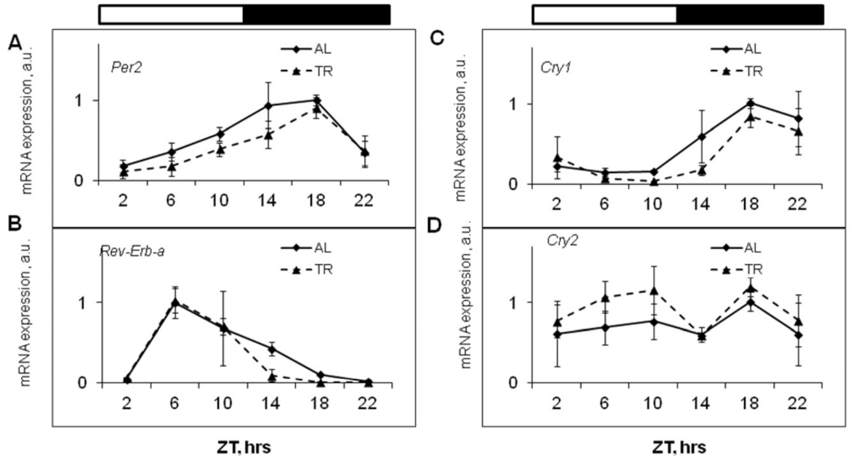

Figure S4. TR does not affect the circadian rhythms in the expression of circadian clock genes. The expressions of circadian clock genes *Per2*, *Cry1*, *Rev-Erb-a* and *Cry2* have been analyzed in the liver of AL (black diamonds, solid black lines) and TR (black triangles, black dashed line) fed mice. Bars on the top of the figure represent light (open bars) and dark (black bars) parts of the day.

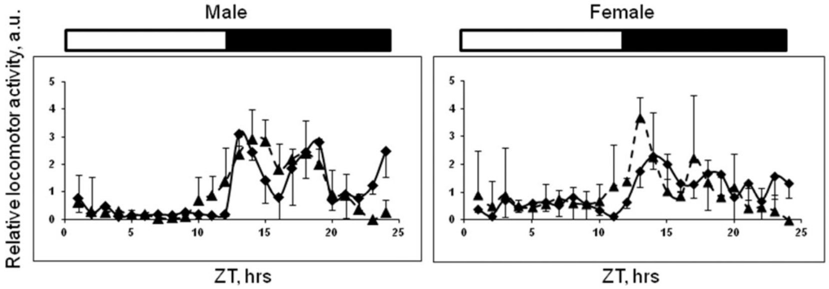

Figure S5 TR does not affect circadian rhythms in behavior. Representative daily in cage activity of male and female mice fed AL (black diamonds, solid black line) or TR (black triangles, black dashed line). Daily behavior of the same mouse was monitored for 4 days on AL diet and for 4 days after 2 weeks of TR. Bars on the top of the figure represent light (open bars) and dark (black bars) parts of the day.
